# Differential methylation of *OPRK1* in borderline personality disorder is associated with childhood trauma

**DOI:** 10.1038/s41380-024-02628-z

**Published:** 2024-06-11

**Authors:** Dorothee Maria Gescher, Denny Schanze, Peter Vavra, Philip Wolff, Geraldine Zimmer-Bensch, Martin Zenker, Thomas Frodl, Christian Schmahl

**Affiliations:** 1 Department of Psychiatry, Psychotherapy and Psychosomatics, Medical Faculty, RWTH Aachen University, Aachen, Germany; 2 Department for General Psychiatry, Center of Psychosocial Medicine, Medical Faculty, Heidelberg University, Heidelberg, Germany; 3 Department of Psychiatry and Psychotherapy, Medical Faculty, Otto-von-Guericke University, Magdeburg, Germany; 4 Institute of Human Genetics, Medical Faculty, Otto-von-Guericke University, Magdeburg, Germany; 5 Department of Biological Psychology, Institute of Psychology, Otto-von-Guericke University, Magdeburg, Germany; 6 Division of Neuroepigenetics, Institute of Zoology (Biology II), RWTH Aachen University, Aachen, Germany; 7 German Center for Mental Health (DZPG), Jena-Magdeburg-Halle, Germany; 8 Center for Intervention and Research on adaptive and maladaptive brain Circuits underlying mental health (C-I-R-C), Jena-Magdeburg-Halle, Germany; 9 Department of Psychosomatic Medicine and Psychotherapy, Central Institute of Mental Health, Medical Faculty Mannheim, Heidelberg University, Mannheim, Germany

**Keywords:** Genetics, Psychiatric disorders

## Abstract

According to a growing body of neurobiological evidence, the core symptoms of borderline personality disorder (BPD) may be linked to an opioidergic imbalance between the hedonic and stimulatory activity of mu opioid receptors (MOR) and the reward system inhibiting effects of kappa opioid receptors (KOR). Childhood trauma (CT), which is etiologically relevant to BPD, is also likely to lead to epigenetic and neurobiological adaptations by extensive activation of the stress and endogenous opioid systems. In this study, we investigated the methylation differences in the promoter of the KOR gene (*OPRK1*) in subjects with BPD (*N* = 47) and healthy controls (*N* = 48). Comparing the average methylation rates of regulatorily relevant subregions (specified regions CGI-1, CGI-2, EH1), we found no differences between BPD and HC. Analyzing individual CG nucleotides (*N* = 175), we found eight differentially methylated CG sites, all of which were less methylated in BPD, with five showing highly interrelated methylation rates. This differentially methylated region (DMR) was found on the falling slope (5’) of the promoter methylation gap, whose effect is enhanced by the DMR hypomethylation in BPD. A dimensional assessment of the correlation between disease severity and DMR methylation rate revealed DMR hypomethylation to be negatively associated with BPD symptom severity (measured by BSL-23). Finally, analyzing the influence of CT on DMR methylation, we found DMR hypomethylation to correlate with physical and emotional neglect in childhood (quantified by CTQ). Thus, the newly identified DMR may be a biomarker of the risks caused by CT, which likely epigenetically contribute to the development of BPD.

## Introduction

Borderline personality disorder (BPD) is a severe clinical condition with pervasive dysfunctions in the perception and regulation of self and interpersonal functioning. Associated with chronic suicidality, non-suicidal self-injury (NSSI), impulsivity, dissociation, and usually high comorbidity such as depression and addiction [1], BPD has a 2.7–5.9% prevalence according to DSM-IV [2, 3]. Its etiology is multidimensional and includes adaptive-developmental psychological factors in addition to the pathobiological aspect of gene and brain function [4, 5]. It has been suggested that the endogenous opioid system (EOS) plays a distinctive role in the core symptoms of BPD [6, 7].

The EOS represents a complex neuromodulatory system, which involves distinctly acting receptor families, including, particularly, the opioid receptors mu (MOR) and kappa (KOR), and their interacting ligands beta-endorphins, enkephalins, and dynorphins. Aside from its central role in pain modulation and stress response, including endocrine and immune functions, the EOS is substantially involved in mood regulation, reward experience, and social attachment processes such as separation-associated distress [8–11].

Stress-induced MOR activation rapidly mediates analgesic and mood-elevating effects, with their basal tonic stimulation being significantly involved in the formation and stability of social bonding and attachment behavior [11]. Acute stress simultaneously leads to CRF-induced dynorphin activation, resulting in KOR-mediated presynaptic inhibition of dopaminergic and serotonergic neurons in the ncl. accumbens, thereby antagonizing the reward system to prevent overexcitation [12, 13]. Overstimulation of the EOS by excessive stress from physical or even social threat, as well as reduced basal opioidergic stimulation, can lead to an adapted receptor expression and an imbalance of the KOR-MOR relationship, resulting in symptoms resembling BPD, such as dysphoria, increased affective irritability and impulsivity, and interpersonal dysfunction [6, 7, 14, 15]. Conversely, opioid antagonists have been shown to reduce BPD-associated symptoms [16, 17] such as NSSI [17], binge eating [17], suicidality [18], and dissociation [19, 20].

A major stress experience and a strong risk factor in the pathogenesis of BPD is early childhood trauma (CT) [21, 22]. Together with genetic predispositions [4, 23], early experiences of unstable and invalidating primary bonding or abuse and neglect impede the formation of a well-integrated personality structure, resulting in impaired personality functioning [24, 25]. These impairments of BPD are only treatable in the long term and can persist chronically [25, 26]. The rigidity of these *experience-driven* difficulties indicates the involvement of acquired biological factors, which may ensue from epigenetic modification.

There is substantial evidence that CT, resulting in exhaustive stimulation of the hypothalamus-pituitary-adrenal (HPA) axis, can induce the adaptation of gene activity via epigenetic modulations such as DNA methylation, and thus leaving a lasting impact of early stress perturbances [27–30]. The glucocorticoid receptor (GR) genes *NR3C1* and *FKBP5*, and particular genes involved in neuronal development and brain plasticity, the humoral stress system and the associated neurotransmitters are substantially involved in early overwhelming stress [31–34]. With genome-wide methylation changes having been found to be associated with CT, its effect appears to be quite widespread [35, 36].

Interestingly, the expression of *OPRK1 (*opioid receptor kappa 1 (KOR)) has also been found to be influenced by CT via different mechanisms, including those that are epigenetic [37]. Surprisingly, very few studies have investigated aspects of the EOS in BPD, with none involving (epi-) genetics, although its pathogenesis, closely related to early life experiences, makes it susceptible to early epigenetic imprinting. Previous methylation studies in BPD have investigated individual regions coding for *BDNF* [38, 39], *NR3C1* [40–44], *FKBP5* [40, 41], *DRD2* [45], *HTR3A*, *HTR2A* [44, 46], *MAOA* and *MAOB* [44], *COMT* [44], and *OXTR* [40]. Some studies have performed genome-wide assays [35, 47], finding the best methylation differences in clusters of functionally related genes to be associated with immune-response, cell-signaling or transcription control [[35, 47, 48], reviewed in [49]].

Inspired by the current state of research compiled here, our study sought to analyze methylation changes of the KOR gene *OPRK1* as a potential candidate with respect to the impact of CT on BPD. We hypothesized that the stress system in BPD subjects was overly activated by CT leading to an epigenetic response in the EOS.

*OPRK1* is located on human chromosome 8q11.2, on the reverse strand [50], and the coding sequence consists of four exons (RefSeq NM_000912) [51]. The *OPRK1* promoter is associated with a CG island (CGI) extending into the beginning of intron 2 (I2). CGI promoters are sparsely methylated and adopt a transcriptionally facilitating status, in which distinct transcription start sites (TSS) can occur [52]. In the *OPRK1* CGI promoter region, three TSS generate distinct transcript variants [51]. In addition, the region contains several transcriptional regulatory elements, e.g., AP-1, AP-2, SP-1, GR, and Ik-1 [51, 53].

In view of the transcriptional and regulatory relevance, we investigated the methylation differences of specific functional segments of the promoter region of *OPRK1* between BPD and HC subjects. The aim of our study was threefold: first, to test for aberrant methylation in BPD of functionally important, a priori specified regions (CGI-1, CGI-2, EH1) within the *OPRK1* promoter, which could result in different gene activity; further, within a 10 kb analysis window in the promoter, to identify differentially methylated CG sites between BPD and HC; second, to exploratorily analyze correlations between CG methylation rates and BPD severity; and third, to investigate the effects of CT on methylation rates.

Therefore, we studied 47 well-characterized females with BPD and compared them with 48 healthy age-matched female controls.

## Methods

### Samples

These novel samples were derived from subjects who took part in different studies within the DFG-funded Clinical Research Unit 256 (KFO256 [4]) and who were recruited between 2012 and 2014 at the Central Institute of Mental Health Mannheim, Germany. The relevant studies were approved by the appropriate Ethics Committee. All participants provided written informed consent including DNA-based analyses.

We selected 47 medication-free, non-smoking female patients with a current diagnosis of BPD and 48 female, non-smoking healthy subjects, matched for age and BMI (Table 1). The general exclusion criteria comprised neurological disorders, current alcohol or drug abuse or dependence in the last 12 months, lifetime diagnosis of schizophrenia, schizoaffective or bipolar disorder, severe medical illness, including cardiac dysregulations, and use of psychotropic medication.

**Table 1:** Descriptive parameters of the study subjects with BPD and HC.

*n* (BPD/HC)	BPD		HC		Group comparison	
	M	SD	M	SD	*t*	*p*
Age (47/48)	25.21	4.21	24.71	3.96	0.602	0.549^a^
BMI (47/48)	24.28	5.58	22.88	3.79	1.423	0.156^a^
BSL-23 (46/48)	2.10	0.76	0.10	0.17	17.483	<0.001^b^
ZAN-BPD (47/48)	1.22	0.43	0.02	0.06	19.011	<0.001^b^
BIS (46/48)	2.36	0.31	1.68	0.28	11.106	<0.001^b^
DES (46/47)	20.61	14.00	2.26	1.80	8.822	<0.001^b^
CTQ (44/48)	2.34	0.66	1.18	0.25	10.997	<0.001^b^

Symptom scales (means): *BSL-23* Borderline Symptom List, *BIS* Barrat Impulsivity Scale, *CTQ* Childhood Trauma Questionnaire, *DES* Dissociative Experience Scale (German version [58]), *ZAN-BPD* Zanarini Rating Scale for BPD.

*M* mean, *SD* standard deviation. Group comparisons were performed with unpaired *t* tests.

^a^two-sided, ^b^one-sided.

The clinical diagnostics for BPD were performed by means of the International Personality Disorder Examination (IPDE [54]), with the Structured Clinical Interview for the DSM-IV (SCID [55]) being used for the exclusion criteria. Further, symptom severity was assessed with the Borderline Symptom List (BSL-23 [56]), the Zanarini Rating Scale for BPD (ZAN-BPD [57]), the adapted German version of the Dissociative Experience Scale (DES) (Fragebogen zu dissoziativen Symptomen (FDS) [58]), and the Barratt Impulsivity Scale (BIS-10 [59]), which includes motor, attentional and nonplanning subscales. Childhood adverse experience was measured using the Childhood Trauma Questionnaire (CTQ [60]). As a standard and widely recognized instrument, the CTQ contains a total score and the five subscales, emotional and physical neglect (EN, PN), and emotional, physical, and sexual abuse (EA, PA, SA). The scales can be evaluated linearly by their score (dimensional evaluation). In addition, cutoff scores can be applied to identify individuals with histories of abuse and neglect (categorical approach). According to the definition by the authors, at least moderate traumatization was classified if mean scores of the different subscales were EA > 2.4, PA > 1.8, SA > 1.4, EN > 2.8, PN > 2.4 [61].

For all questionnaires used (BSL-23, ZAN-BPD, DES, BIS-10, CTQ), we calculated with mean scores.

### DNA methylation analysis

Analyses were performed on isolated genomic DNA from whole blood samples of each subject. DNA concentration was measured and adapted by using a Qubit^TM^ 4 fluorometer (Invitrogen^TM^, Carlsbad, CA, USA). For preparation, DNA was sheared into fragments of 150–200 bp by focused-ultrasonic fragmentation using the Covaris S220 sonication system (Covaris Ltd, Brighton, GB). DNA methylation was analyzed via multiplex bisulfide PCR sequencing in accordance with the manufacturer’s instructions without modification (SureSelect^XT^ Methyl-Seq Target Enrichment System for Illumina Multiplexed Sequencing, Agilent Technologies Inc., Santa Clara, CA, USA).

Sequencing was done on an Illumina MiSeq^TM^ System (2 × 150-bp paired-end) (Illumina Inc., San Diego, CA, USA) followed by demultiplexing and quality control for the generated reads (Q30 > 90%).

### Target regions of *OPRK1*

**Fig. 1:**
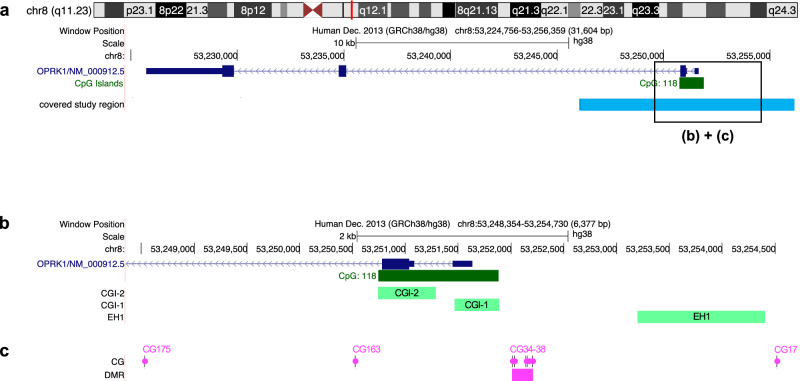
Localization of studied gene regions in *OPRK1.* **a** Gene structure of *OPRK1* and covered 10 kb-target section, sequenced in this study. **b** Localization of a priori specified functional gene regions EH1, CGI-1 and CGI-2. **c** Localization of in BPD differentially methylated CG sites and DMR (CG34-CG38), respectively.

For the designing of target regions in *OPRK1*, localization of exons, TSS, promoter and known gene regulation elements (GRE) were identified using UCSC (https://genome.ucsc.edu) and ensembl genome browser (https://www.ensembl.org) databases. All gene localizations in this article refer to hg38. The gene target region to be studied was set from 5 kb upstream to 5 kb downstream from the TLS (chr8:53251036, [51]), comprising a distance from chr8:53246080-53256098 including 189 CG sites. It starts 4.448 kb 5’-upstream from exon 1 and spans to 4.695 kb within intron 2 (Fig. 1a). This defined region constituted part of a customized panel (SureSelect^XT^ targeted methyl panel, Agilent Technologies, Inc., CA, USA).

For individual analyses of functionally relevant sites, three regions of interest were defined. CG-island 1 (CGI-1): chr8:53251468-53251883 (including 52 CG, 416 bp), CG-island 2 (CGI-2): chr8:53250744-53251285 (including 62 CG, 542 bp), and enhancer (EH1): chr8:53253201-53254400 (including 4 CG, 1.19 kb). These defined sections are all located in the promoter chr8:53250800–53252001 (GH08J053251, https://genome.ucsc.edu).

EH1 is located 1.2 kb upstream from the promoter and 1.564 kb upstream of exon 1, respectively, while CGI-1 and CGI-2 are located closely together within the promoter region. Thus, CGI-1 starts 246 bp upstream of exon 1 and extends 169 bp inside. CGI-2 begins 162 bp downstream from the start of intron 1 and extends 341 bp into intron 2 (Fig. 1b).

### Bioinformatics

Generated fastq files were quality-checked by means of fastqc v0.11.9 [62]. Adapters were trimmed using TrimGalore v0.6.7 [63] and subsequently analyzed with Bismark v0.23.1 [64] using the embl hg38 primary assembly DNA (GenBank Assembly ID GCA_000001405.28) for reference genome to extract the number of converted and unconverted cytosine for each CG in the targeted genomic region. Statistical analyses were programed and carried out with R (version 4.3.0; R Core Team, 2023). All cytosines with a coverage less than 25 reads were excluded from calculations.

### Statistical analysis

In the first part of the study, we focused on methylation differences between patients and healthy individuals. For the first analysis, we assessed the a priori defined target regions (CGI-1, CGI-2, and EH1). First, we calculated, for each subject separately, the mean methylation rate per target region by averaging the methylation rates of all CG sites within that target region. Second, we sought to determine whether these average methylation rates differed between patients and healthy individuals by modeling the mean methylation rate as a function of group (BPD vs HC), age and BMI in a regression model, and testing the significance by the *t*-value associated with the difference between BPD and HC. We assessed the prerequisites (notably normality and homoscedasticity of residuals) of all parametric regressions using Shapiro-Wilk and Breusch-Pagan tests. Only for one analysis (the defined target region CGI-2) the normality assumption was violated, while heteroscedasticity was never detected. To assess the robustness of our parametric results, we recalculated this regression using a bootstrapping algorithm (with 10,000 replicates), which gave similar results to the parametric version (including a non-significant difference in methylation rates between the two groups). Hence, we report in the remaining manuscript only the parametric results. As regards the descriptive values, we report the estimated mean methylation rates at the mean age and mean BMI.

To gain a more detailed view, we then assessed single CG sites’ methylation rates for the entire sequenced gene region of *OPRK1* (189 CG) in this study. To account for different coverage of individual CG’s, we performed logistic regression for all analyses, again controlling for age and BMI, which resulted in 174 individual CG sites with enough data. We tested significance with the *t*-value of the difference in log-odds between BPD and HC. To control for multiple comparisons, we set the significance level at p 0.05 with an FDR correction for multiple testing [65].

Based on the results of this analysis, we defined a novel differentially methylated region (DMR) located on chromosome 8 (chr8:53252014-53252198). To assess the consistency in methylation rates of these CG sites, we calculated correlations across participants. Then, analogous to the a priori defined target regions, we calculated the DMR’s mean methylation rate by averaging the methylation rate of each CG site. As done for the a priori regions, we assessed group differences using a regression, again controlling for age and BMI. Note that this approach does not account for the potentially different coverage across the individual CGs and across subjects, and, therefore, the methylation rate may not be significantly different between BPD and HC, even though each CG site (with the statistically more powerful analysis above) is.

In the second part of the study, we aimed to quantify the relationship between the methylation rate and symptom severity of this novel DMR. To that end, we estimated a series of regression models, in which we modeled the mean methylation rate of the DMR as a function of the linear symptom scales BSL-23, ZAN-BPD, DES, and BIS, controlling for age and BMI. The resulting regression slope between symptom scale and methylation rate is similar to a correlation, also with age and BMI having been controlled for.

In the third analysis, we assessed the influence of childhood trauma, as estimated with the CTQ, on methylation levels of the novel DMR. Analogous to the symptom severity, we modeled the methylation rate as a function of CTQ severity, again controlling for age and BMI, in a regression model.

## Results

### The average methylation rates of a priori specified subregions CGI-1, CGI-2 and EH1 do not differ in BPD

We found no significant differences in the average methylation of the a priori defined target regions CGI-1 (mC = 4.2%, and 4.5% in BPS and HC, respectively; difference: *t* (90) =−1.78, *p* = 0.08), nor in that of CGI-2 (mC = 4.1%, and 4.2% in BPS and HC, respectively; difference: *t* (90) = −1.35, *p* = 0.18) and EH1 (mC = 52.8%, and 52.3% in BPS and HC, respectively; difference: *t* (91) = 0.87, *p = 0.39*).

### Methylation rates of eight distinct CG sites within the *OPRK1* promoter and intron 2 are reduced in BPD

Within the total of 174 analyzed CG sites across the *OPRK1* gene, we found eight CG sites to be methylated significantly differently between BPD and HC. Of these eight sites, six are located within the promoter and two within intron 2 (Table 2). As the most upstream site, CG17 is located directly before EH1. The next five CGs downstream are adjacent to one another and are located exactly 5’ ahead of CGI-1 (CG34, CG35, CG36, CG37, CG38). CG163 and CG175 are located 255 bp and 2.251 kb downstream from the start of intron 2, respectively (Fig. 1c).

**Table 2:** Methylation rates of individual CpG sites within *OPRK1* that differ significantly between subjects with BPD and HC, significance level at *p* 0.05 with an FDR-correction for multiple testing (Benjamini and Hochberg).

CG ID	Position (hg38)	Localization (*OPRK1*)	BPD methylation rate [%]	HC methylation rate [%]	Odds-Ratio	*t*-value	df	*p*-value (adjusted)
CG17	chr8:53254514	Promoter	92.28	95.72	1.870	10.2	91	0.0000
CG34	chr8:53252198	Promoter	54.24	56.63	1.100	3.56	91	0.0161
CG35	chr8:53252152	Promoter	53.29	55.47	1.090	3.12	91	0.0395
CG36	chr8:53252137	Promoter	66.71	68.80	1.100	3.19	91	0.0354
CG37	chr8:53252029	Promoter	60.29	62.91	1.120	3.72	91	0.0161
CG38	chr8:53252014	Promoter	54.24	56.59	1.100	3.19	91	0.0354
CG163	chr8:53250525	Intron 2	33.75	36.62	1.130	3.56	91	0.0161
CG175	chr8:53248529	Intron 2	82.76	81.17	0.898	−3.35	91	0.0284

The methylation rates of CG34 to CG38 were highly correlated with each other (Supplementary Table S1, Supplementary Fig. S1). On this basis, and due to the adjacent localization of CG34-CG38, we defined this cluster for further analyses as a differently methylated region ((DMR), 184 bp). When mirroring the analysis for the a priori defined regions, we found that the mean methylation rate of this DMR was significantly smaller for the BPD group (57.9%) than for the HC group (59.9%; difference: 2.04 ± −0.98, *t* (91) = −2.08, *p* = 0.04).

### Methylation rate of the *OPRK1* DMR is related to BPD symptom severity

We found the methylation rates of the novel DMR to decrease with the overall BPD symptom severity (as measured by BSL-23: *b* = −0.986, *p* = 0.024), and with trait impulsivity (as measured by BIS: *b* = −2,32, *p* = 0.04), including its motor subcomponent (as measured by the motor subscale of BIS: *b* = −1.91, *p* = 0.4). Notably, the other symptom scales (DES, ZAN-BPD) also showed a negative relationship with the mean methylation rate of the novel DMR (consistent with a difference between BPD and HC) but did not reach significance (all other *p*s > 0.065) (Supplementary Table S2). The analyses didn’t reach significance for the groups (HC and BPD) separately.

### Childhood neglect is related to the methylation rate of *OPRK1* DMR

Finally, we observed a significant decrease in the methylation rate of the novel DMR in the presence of higher levels of emotional neglect (as measured by the dimensional measure CTQ, subscale EN (CTQ_EN: *b* = −0.79, *p* = 0.044)), with the overall CTQ score showing a similar trend (CTQ: *b* = −1.31, *p* = 0.054). When re-running the analysis with the categorical approach of the CTQ, we observed significantly lower methylation rates for emotionally (CTQ_ENcat: *b* = −2.23, *p* = 0.037) and physically neglected subjects (CTQ_PN: *b* = −2.50, *p* = 0.036) (Supplementary Table S3).

## Discussion

Our study analyzed DNA methylation of the *OPRK1* in subjects suffering from BPD and in HC. We identified three solitary CG sites within the promoter and intron 2, respectively, and five adjacent CG sites within the promoter region of *OPRK1*, with a significant methylation difference between the BPD and HC groups. The five adjacent CG dinucleotides constituted a DMR that was located directly upstream of CGI-1 and CGI-2, two regions that, as part of the CGI promoter, are intrinsically poorly methylated (methylation gap) to enable transcription [53].

**Fig. 2:**
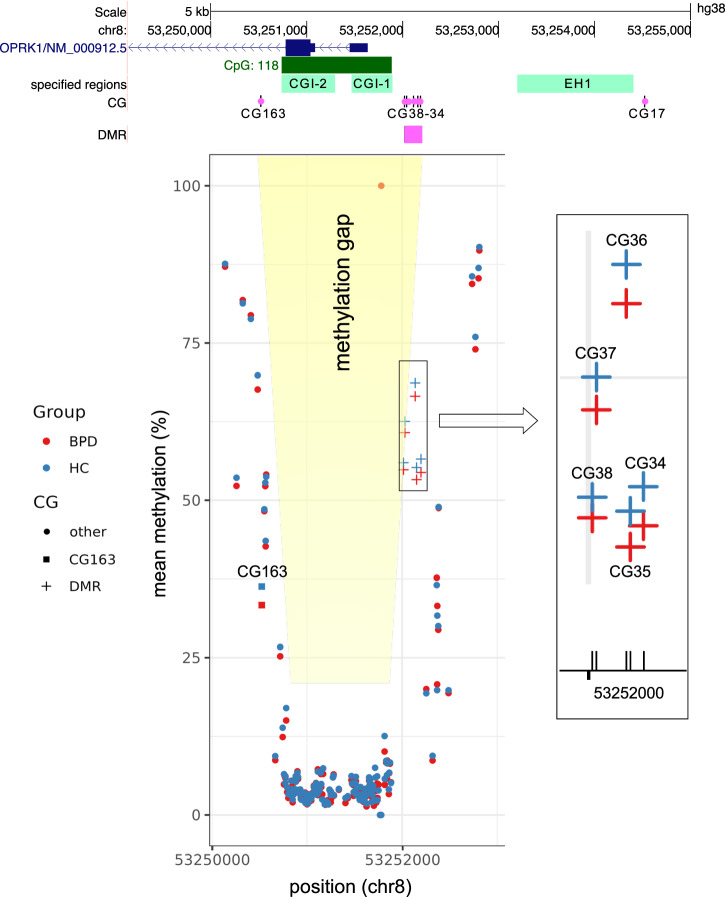
Mean methylation rates of individual CG nucleotides within the promoter region of *OPRK1* (subregion chr8:53250000-53253000) and localization of CG163 and DMR (CG34-CG38), which are more poorly methylated in BPD. Both the new DMR localizing within the falling slope (5’) and CG163 within the rising slope (3’) of the methylation gap strengthen the effect of the promoter hypomethylation in BPD. Detail: enlarged view of the clustering CG34-38, each hypomethylated in BPD, building the DMR. Red dots: BPD, blue dots: HC.

All the differentially methylated solitary CG sites and the DMR revealed significantly decreased methylation rates in BPD. Thus, the DMR location in the falling slope (5’), and conversely that of CG163 in the rising slope (3’) of the methylation gap, result in an increased steepness of methylation changes in BPD, with the gap presumably facilitating gene transcription (Fig. 2).

Functionally relevant gene regions such as CGI-1, CGI-2 and EH1, however, did not differ significantly in their average methylation between BPD and HC.

Methylation in the promoter region had previously been shown to control transcription of the *OPRK1* [66]. The hypomethylated DMR in the *OPRK1* promoter may therefore lead to overexpression of *OPRK1*. KOR overactivity mediates the effects of chronic stress on dysphoria and anhedonia [7, 14], which is well explained by the inhibitory effect of the KOR/dynorphin system on dopaminergic neurons in the mesolimbic reward system [12, 13, 67]. Drug addiction, risk- and attention-seeking behavior, eating disorders, and NSSI can be understood as desperate attempts to overcome anhedonia and endorphin depletion through stimulation of the EOS and the reward system [6, 68]. In a recent study, a specific KOR antagonist has been found to effectively reduce anhedonia in depressed patients [69].

In our study, the subjects’ symptom severity, as measured by the BSL-23, was found to be significantly associated with the degree of DMR *hypo*methylation, strengthening the link between BPD and methylation level. Furthermore, this approach mirrors the dimensional view and the concept of continuous trait expression in personality disorder according to the Alternative Model for Personality Disorders in DSM-5 Section III (APA 2013, [70]).

Thus, our findings of hypomethylation within the *OPRK1* promoter are both in line with and add to the current understanding of the interplay of opioid receptors. They support the notion of a chronic KOR overactivity being related to BPD.

However, either overstimulation of KOR or under-stimulation of MOR can drive a KOR-MOR imbalance, both being seminal in their individual effects in relation to BPD symptomatology, while at the same time interacting inextricably.

With respect to our third objective, we found a negative association between the average DMR methylation rate in the *OPRK1* promoter and childhood emotional and physical neglect. Characteristically, persistent neglect by primary attachment figures can have pervasive consequences such as chronic basal under-stimulation of the mu-opioid system [6, 71], which, being responsible for social motivation, mediates the hedonic aspects of social reward, touch, attention, and affection [71–73]. Indeed, patients with NSSI were found to have reduced serum ß-endorphin levels [74–76]. Consistent with these findings, in a PET study of BPD patients, Prossin and colleagues [15] found a likely compensatory upregulation of the central MORs in relation to an increased activation following experimental provocation of negative affectivity. It can be speculated that this MOR hypersensitization with hyperexcitation may in turn provoke a strong KOR counter-activation, eliciting an increased KOR expression. The decreased DMR methylation seen in our BPD sample, which may be an adaptation to early environmental conditions, is therefore in line with this hypothesis.

As we did not study the MOR encoding of the *OPRM1* gene, we cannot comment on the KOR/MOR expression ratio. In principle, high KOR expression can also be led by repeated high stress levels, which trigger increased MOR or CRF activity with consequent KOR activation to dampen the overall stress system. However, our overall results suggest that experiences of childhood neglect are particularly related to *OPRK1* methylation changes and correlate with BPD symptomatology.

There is a paucity of research on epigenetic alterations in the *OPRK1* in psychopathology and childhood trauma, with no studies, to our knowledge, involving BPD. *Hyper*methylation in the promoter has been linked to heroin addiction [66], and *hyper*methylation within the intron 2 is associated with CT [37]. This, along with our findings regarding promoter *hypo*methylation in BPD, suggests that heroin addiction and BPD lie at different poles of the opioid system imbalance.

As regards the limitations of the study, we examined the methylation of only one isolated gene, *OPRK1*, whose activity is embedded in a complex interplay with the MOR, the endogenous opioids, and the humoral stress system. Thus, a full analysis of the EOS dysfunctions would require a broader view, which has not yet been taken at the epigenetic level for BPD despite its intrinsic etiological links to external factors including childhood adversity. Moreover, there is evidence from post-mortem studies that the association between CT and *OPRK1* mRNA expression differs depending on the brain regions [37]. Therefore, no general functional conclusions with respect to psychopathology and patho-etiology can be drawn based on gene transcription. Nevertheless, we demonstrated that aberrant methylation of a DMR within the promoter of *OPRK1* in peripheral blood cells may be an indicator of BPD and its symptom severity, and that it is associated with CT at the sublevel of emotional and physical neglect.

The diagnosis of BPD is associated with a substantial number of genetic variants (e.g. in *FKBP5* [77], *CRHR1* [23], *COMT* [78, 79], *TPH2*, *HTR2A*, *SLC6A4*, *MAOA*, *SLC6A3* [80]) which are also differentially sensitive to CT [23, 80–82]. But, in our analyses, we did not statistically control for any influence of these genetic variants on our methylation analyses of *OPRK1* given that there is no known interaction between these genes and the *OPRK1* methylation rate. Positing any such interaction, therefore, would be purely speculative, forcing us to control for all genes theoretically associated with BPD (without any relevant evidence thereof), and thus overstraining the statistical power of our cohort without a valid rationale.

As for the dimensional aspect of our investigations, the study cohort was not optimally suited given that it consisted of a group of healthy individuals without psychopathology and patients with full-blown BPD, thus lacking an intermediate group with lower BPD levels. Finally, the study was conducted with samples from only 95 subjects, of whom 47 were with BPD. The results must be replicated in a larger cohort to strengthen the evidence.

To sum up, our data indicate a mechanism through which childhood neglect may contribute to the development of BPD. If our preliminary findings can be replicated in larger samples, the level of methylation of an indicative region, such as the newly identified DMR, may be evaluated as a feature of a risk constellation for BPD. It may also act as an effective marker for early detection or treatment efficacy. Based on our findings, increased KOR expression may explain chronic anhedonia, suicidality, or inner emptiness in BPD, which may be a trigger for self-injurious and risk-seeking behavior and drug use. A selective KOR antagonist has recently been found to reduce anhedonia in patients with depression and anxiety [69]. This suggests that such receptor-selective agents ought to be tested for the treatment of BPD, highlighting the KOR receptor as a promising pharmacological target. For a fundamental understanding of the EOS in the pathogenesis and maintenance of BPD, including its therapy, the receptors involved in the EOS should be conceptualized as a functional ensemble in further research.

## Supplementary information

Supplemental material

## Data Availability

The data that support the findings of the current manuscript have been made available within the paper and its supplementary figures/tables as far as possible under data protection law. Additional deidentified data is available from the corresponding author on reasonable request.
