# Supplementary material for: Differential methylation of OPRK1 in borderline personality disorder is associated with childhood trauma
Source: Mol Psychiatry. 2024 Jun 11;29(12):3734–41. doi: 10.1038/s41380-024-02628-z (PMC11609100; doi:10.1038/s41380-024-02628-z)
Supplement: Supplementary file 1 — Supplemental material [file 41380_2024_2628_MOESM1_ESM.pdf]

SUPPLEMENTAL MATERIAL

Differential methylation of *OPRK1* in borderline personality disorder is associated with childhood trauma

Gescher DM, Schanze D, Vavra P, Wolff P, Zimmer-Bensch G, Zenker M, Frodl T, Schmahl C.

Table S1. Intercorrelation of CG34-CG38 constituting the DMR.

Figure S1. Intercorrelation of CG34-CG38 constituting the DMR

Table S2. Regression analysis of the DMR with BPD symptom scales and subscales, corrected by age and BMI.

Table S3. Regression analysis of the DMR with CTQ total and subscales, corrected by age and BMI.

Figure S2. Regression analysis of the DMR with CTQ and BPD symptom scales and subscales, corrected by age and BMI (scatterplots).

Table S4. Read counts and mean methylation rates per covered CG.

Table S1. Intercorrelation of CG34-CG38 constituting the DMR.

	**CG34**	**CG35**	**CG36**	**CG37**	**CG38**
	r (*p*)	r (*p*)	r (*p*)	r (*p*)	r (*p*)
**CG34**	1 *(N/A)*	0.5069 (<*.0001*)	0.4632 (<*.0001*)	0.4089 (<*.0001*)	0.2845 (*.0052*)
**CG35**		1 *(N/A)*	0.6703 (<*.0001*)	0.4553 (<*.0001*)	0.3211 (*.0015*)
**CG36**			1 *(N/A)*	0.3828 (*.0001*)	0.1497 (*.1477*)
**CG37**				1 *(N/A)*	0.4199 (<*.0001*)
**CG38**					1 *(N/A)*

R Pearson's correlation coefficient, *p* p-value.

Figure S1. Intercorrelation of CG34-CG38 constituting the DMR. (Pearson's correlation coefficients)

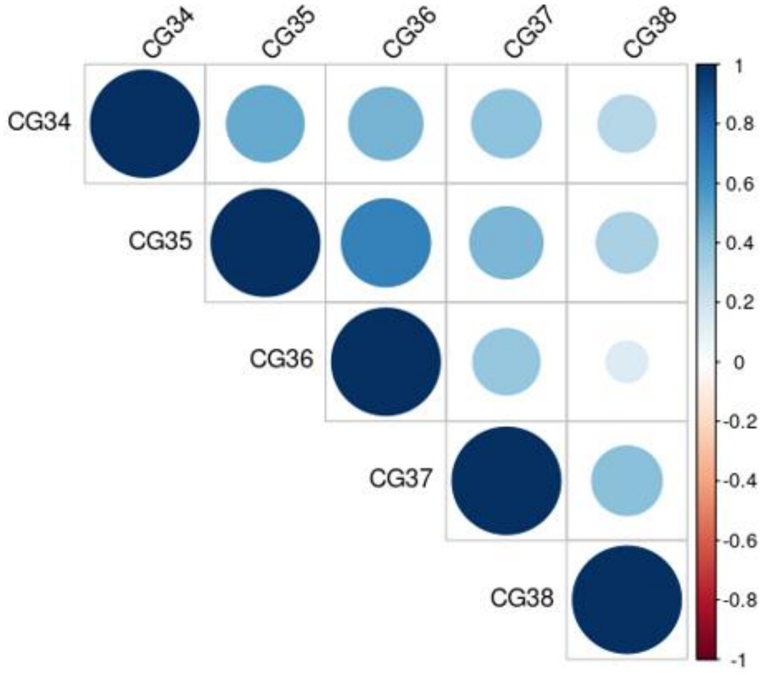

Table S2. Regression analysis of the DMR with BPD symptom scales and subscales, corrected by age and BMI.

**scale**	**beta**	**t_value**	**df**	***p-value***
**BSL**	**-0.986**	**-2.301**	**90**	***.024****
**BIS_total**	**-2.324**	**-2.058**	**90**	***.042****
**BIS_motor**	**-1.914**	**-2.115**	**89**	***.037****
BIS_attentional	-1.680	-1.857	90	*.067*
BIS_nonplanning	-1.466	-1.156	90	*.251*
DES	-0.07	-1.872	89	*.065*
ZAN-BPD	-0.133	-1.606	91	*.112*
ZAN_aff	-0.348	-1.787	91	*.077*
ZAN_cogn	-0.305	-1.182	91	*.24*
ZAN_imp	-0.191	-0.396	91	*.693*
ZAN_int	-0.534	-1.614	91	*.11*

Symptom scales (means): BSL Borderline Symptom List, BIS Barrat Impulsivity Scale, BIS_motor Motoric Subscale of BIS, BIS_attentional Attentional Subscale of BIS, BIS_nonplanning Nonplanning Subcale of BIS, DES Dissociative Experience Scale (German version [58]), ZAN-BPD Zanarini Rating Scale for BPD, ZAN_aff Affect Sector of ZAN-BPD, ZAN_cog Cognition Sector of ZAN-BPD, ZAN_imp Impulsivity Sector of ZAN-BPD, ZAN_int Interpersonal Sector of ZAN-BPD.

Table S3. Regression analysis of the DMR with CTQ total and subscales, corrected by age and BMI.

**Scale**	**slope b**	***t*-statistic**	**df**	***p*-value**
CTQ_total	-1.308	-1.955	88	*.054*
CTQ_EA	-0.768	-1.968	88	*.052*
CTQ_PA	-1.307	-1.791	88	*.077*
CTQ_SA	-0.137	-0.16	89	*.873*
**CTQ_EN**	**-0.790**	**-2.047**	**88**	***.044****
CTQ_PN	-0.932	-1.222	88	*.225*
CTQ_EAcat	-1.597	-1.510	88	*.135*
CTQ_PAcat	-2.266	-1.404	88	*.164*
CTQ_SAcat	1.131	0.906	88	*.367*
**CTQ_ENcat**	**-2.233**	**-2.119**	**88**	***.037****
**CTQ_PNcat**	**-2.495**	**-2.129**	**88**	***.036****
CTQ_trauma	-1.716	-1.697	88	*.093*
CTQ_trauma_n	-0.591	-1.773	87	*.080*

CTQ_total: CTQ total score (means), CTQ subscales (means): EA emotional abuse, PA physical abuse, SA sexual abuse, EN emotional neglect, PN physical neglect. EAcat, PAcat, SAcat, ENcat, PNcat: EA, PA, SA, EN, PN categorial assessment of CTQ subscales. Categorial assessment of CTQ subscales: traumatization was classified if mean scores of subscales were EA>2.4, PA>1.8, SA>1.4, EN>2.8, PN>2.4 (according to Bernstein & Fink, 1998 [61]). CTQ_trauma: criterion met if at least one subscale categorically fulfilled. CTQ_trauma_n: number of categorically fulfilled subscales.

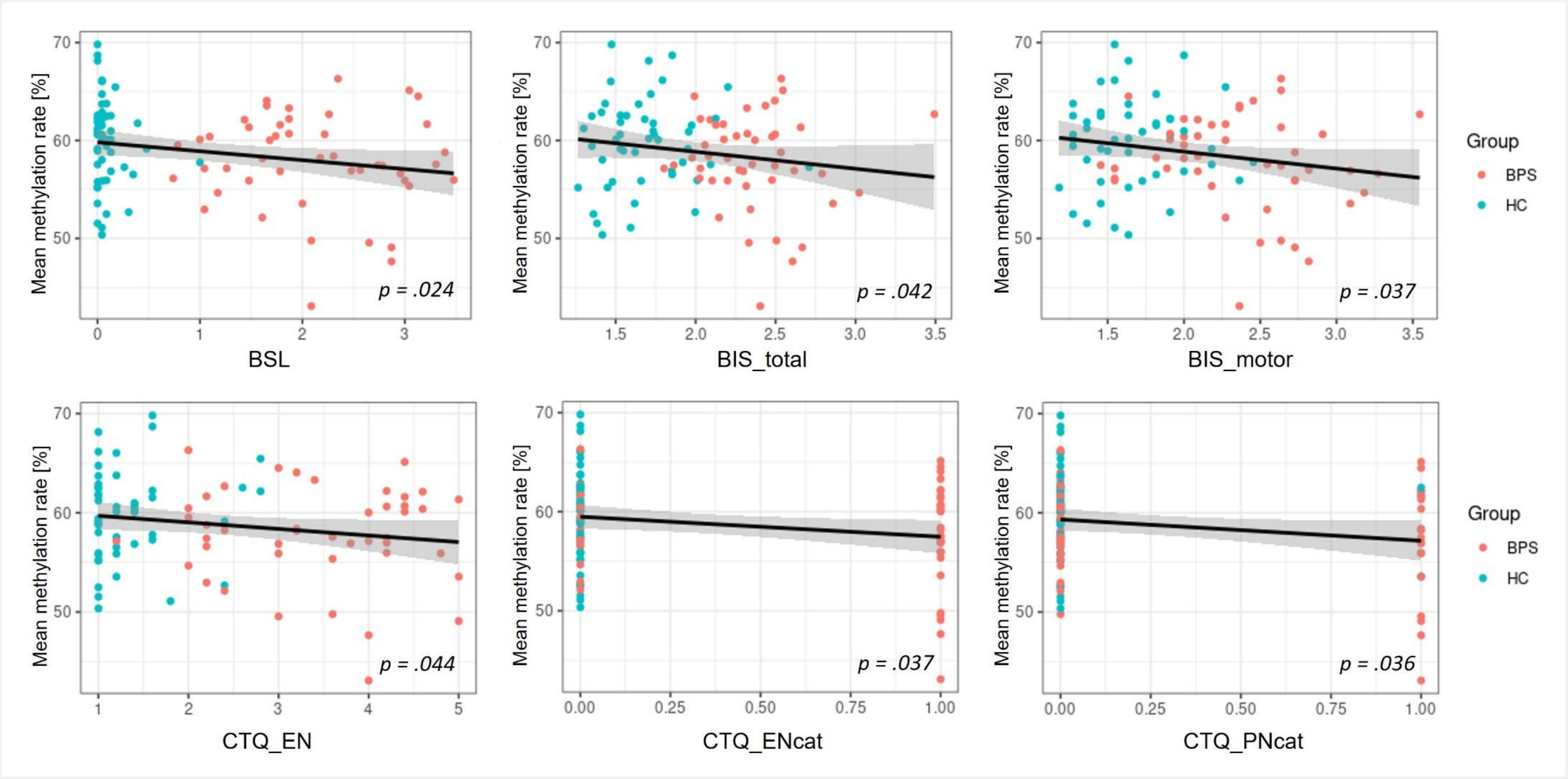

Figure S2. Regression analysis of the DMR with CTQ and BPD symptom scales and subscales, corrected by age and BMI (scatterplots). Mean scores: BSL Borderline Symptom List, BIS Barrat Impulsivity Scale, BIS_motor Motoric Subscale of BIS. CTQ_EN CTQ emotional neglect subscale, CTQ_ENcat/ CTQ_PNcat: categorial assessment of CTQ emotional neglect (EN) and physical neglect (PN) subscales. Cyan dots: healthy subjects, red dots: BPD subjects. Gray colored area: 95% confidence interval.

Table S4. Read counts and mean methylation rates per covered CG.

CG ID	position (chr8)	read count mean	read count median	read count minimum	read count maximum	mean methylation [%]	mean methylation [%] BPS	mean methylation [%] HC
CG1	53255932	188.99	189	29	307	87.27	86.98	87.56
CG2	53255847	168.44	174	31	272	91.00	90.67	91.32
CG3	53255505	100.64	101	22	177	94.46	94.05	94.87
CG4	53255462	134.53	139	27	230	94.20	93.93	94.46
CG5	53255423	176.51	182	40	287	82.61	82.22	82.98
CG6	53255371	222.89	229	47	356	83.63	83.39	83.88
CG7	53255252	192.71	199	43	306	81.49	81.69	81.30
CG8	53254989	158.16	162	39	248	79.16	78.55	79.75
CG9	53254978	154.12	154	41	249	82.34	81.94	82.73
CG10	53254963	147.46	145	35	237	80.71	80.92	80.50
CG11	53254958	145.37	144	36	236	69.47	69.00	69.92
CG12	53254863	142.87	144	29	218	20.31	20.61	20.01
CG13	53254790	156.95	158	31	259	81.48	81.29	81.67
CG14	53254714	186.34	188	43	296	83.64	83.44	83.84
CG15	53254561	213.05	221	40	330	78.31	77.87	78.73
CG16	53254516	206.80	216	35	338	95.42	95.24	95.60
CG17	53254514	205.95	215	38	335	93.39	91.77	94.98
CG18	53254249	140.39	143	32	205	37.07	36.62	37.51
CG19	53253985	193.17	198	57	304	85.98	85.47	86.48
CG20	53253619	156.05	162	28	256	57.65	58.76	56.57
CG21	53253155	206.76	206	40	346	29.43	30.24	28.65
CG22	53252812	164.01	170	33	298	90.00	89.72	90.27
CG23	53252804	166.40	168	33	296	86.11	85.28	86.93
CG24	53252771	149.51	154	25	259	75.00	74.00	75.97
CG25	53252736	137.32	143	17	243	85.00	84.39	85.59
CG26	53252488	167.73	171	28	252	19.59	19.37	19.81
CG27	53252384	179.69	181	28	284	48.86	48.79	48.93
CG28	53252380	179.37	181	29	275	29.72	29.42	30.02
CG29	53252370	181.18	184	30	282	32.44	33.23	31.68
CG30	53252368	181.69	183	29	283	20.29	20.75	19.83
CG31	53252364	184.66	187	29	292	37.12	37.7	36.54
CG32	53252320	211.32	213	33	325	9.03	8.65	9.40
CG33	53252254	234.74	239	49	375	19.66	20.00	19.33
CG34	53252198	234.71	240	51	385	55.52	54.44	56.57
CG35	53252152	219.32	223	48	367	54.27	53.29	55.23
CG36	53252137	216.97	221	47	361	67.62	66.54	68.67
CG37	53252029	201.56	207	54	324	61.65	60.75	62.54
CG38	53252014	202.43	204	53	328	55.43	54.86	55.99
CG39	53251883	105.84	106	24	173	5.14	5.18	5.09
CG40	53251872	95.03	94	23	157	8.24	8.14	8.33

CG41	53251864	86.04	86	22	139	6.67	6.63	6.71
CG42	53251860	80.67	80	22	131	3.71	3.34	4.07
CG43	53251844	57.58	58	12	98	8.41	8.64	8.19
CG44	53251840	48.81	48	10	84	6.05	5.78	6.32
CG45	53251832	36.69	36	8	63	8.47	8.32	8.61
CG46	53251817	14.72	15	4	28	5.27	4.80	5.72
CG47	53251815	10.55	10	3	19	11.33	10.08	12.55
CG48	53251780	1	1	1	1	33.33	100.00	0
CG49	53251776	1	1	1	1	0	0	0
CG50	53251772	1.17	1	1	2	0	0	0
CG51	53251767	1.21	1	1	3	0	0	0
CG52	53251751	1.94	2	1	5	4.4	4.82	3.92
CG53	53251741	2.71	3	1	7	2.55	2.71	2.38
CG54	53251737	3.19	3	1	9	5.53	4.96	6.12
CG55	53251728	4.70	4.5	1	12	2.15	1.99	2.30
CG56	53251719	5.94	6	1	17	4.88	3.82	5.95
CG57	53251715	6.82	7	1	20	5.95	4.38	7.49
CG58	53251702	10.00	10	1	28	1.93	1.48	2.38
CG59	53251694	12.60	12	2	32	3.53	2.40	4.64
CG60	53251692	13.13	13	2	33	3.33	2.62	4.03
CG61	53251687	14.92	15	2	36	3.07	2.94	3.21
CG62	53251681	16.77	16	2	41	2.09	2.11	2.06
CG63	53251676	19.06	19	3	42	3.48	3.00	3.96
CG64	53251673	19.15	20	3	42	4.29	4.86	3.74
CG65	53251667	24.15	24	4	47	4.39	4.79	3.99
CG66	53251662	27.67	28	5	55	5.14	4.77	5.51
CG67	53251657	31.59	31	5	65	4.95	4.63	5.26
CG68	53251652	36.27	35	5	81	5.7	5.44	5.96
CG69	53251644	44.13	43	7	91	3.82	3.31	4.31
CG70	53251641	47.54	47	7	93	3.96	3.59	4.32
CG71	53251625	64.18	63	14	123	1.6	1.37	1.83
CG72	53251621	68.16	68	14	129	3.07	2.98	3.16
CG73	53251611	80.89	80	18	150	4.15	4.15	4.15
CG74	53251602	90.01	88	19	161	2.5	2.56	2.43
CG75	53251598	96.60	93	20	169	2.17	2.08	2.26
CG76	53251595	102.43	103	20	178	2.32	2.38	2.25
CG77	53251580	125.07	125	23	226	3.3	3.3	3.29
CG78	53251576	130.66	130	23	232	2.58	2.32	2.84
CG79	53251569	141.35	141	24	255	2.58	2.46	2.69
CG80	53251563	148.61	148	27	267	4.14	3.85	4.42
CG81	53251556	159.74	159	28	285	2.55	2.70	2.41
CG82	53251529	187.42	191	33	333	3.38	3.38	3.38
CG83	53251522	189.52	191	35	336	3.32	3.49	3.15
CG84	53251516	189.79	192	35	331	5.70	5.36	6.04
CG85	53251514	189.34	193	36	331	3.75	3.49	4.00
CG86	53251512	189.17	192	36	327	5.01	5.03	4.99

CG87	53251492	185.37	187	32	322	3.35	3.06	3.64
CG88	53251487	183.16	186	30	326	4.91	4.79	5.02
CG89	53251475	177.61	181	30	317	5.02	5.07	4.97
CG90	53251469	171.12	175	30	310	5.85	5.53	6.16
CG91	53251431	119.82	120	24	225	2.85	2.69	3.00
CG92	53251406	83.78	83	16	159	2.32	1.91	2.72
CG93	53251336	24.09	23	6	46	4.09	4.02	4.16
CG94	53251285	38.56	38	7	72	3.13	3.09	3.17
CG95	53251281	41.34	41	7	77	6.34	6.44	6.24
CG96	53251274	44.67	44	9	82	5.97	5.99	5.95
CG97	53251254	51.61	52	11	91	3.13	2.55	3.70
CG98	53251251	52.36	52	11	92	2.60	2.01	3.18
CG99	53251249	53.41	54	13	98	3.69	3.75	3.63
CG100	53251238	56.64	58	13	102	4.02	4.00	4.05
CG101	53251227	62.16	62	16	106	1.70	1.72	1.69
CG102	53251218	66.78	67	15	111	2.16	2.35	1.97
CG103	53251198	69.34	68	13	108	2.12	2.00	2.24
CG104	53251195	69.56	70	13	110	1.67	1.63	1.72
CG105	53251188	70.56	69	13	111	1.95	2.21	1.69
CG106	53251183	71.33	70	13	111	2.24	2.28	2.20
CG107	53251171	73.32	71	13	117	6.96	6.50	7.40
CG108	53251162	75.25	73	13	124	2.87	3.29	2.47
CG109	53251151	76.68	76	15	127	4.68	4.46	4.90
CG110	53251148	76.89	75	16	124	6.61	6.49	6.72
CG111	53251136	78.93	78	19	127	4.35	4.34	4.37
CG112	53251134	79.43	79	18	125	4.08	4.26	3.90
CG113	53251115	86.01	86	22	131	7.05	7.24	6.88
CG114	53251113	87.34	88	22	130	3.3	3.17	3.43
CG115	53251105	89.51	91	20	139	3.43	3.58	3.28
CG116	53251096	90.14	92	19	139	5.04	5.07	5.02
CG117	53251090	92.21	94	21	144	3.37	3.30	3.44
CG118	53251085	93.06	95	21	143	3.30	3.31	3.29
CG119	53251082	93.45	95	21	143	3.94	3.63	4.24
CG120	53251073	94.48	96	23	149	4.53	4.62	4.45
CG121	53251063	95.22	97	25	157	3.52	3.58	3.47
CG122	53251046	94.37	95	25	149	2.46	2.32	2.60
CG123	53251027	92.16	93	24	156	2.23	2.14	2.32
CG124	53251013	90.14	93	22	150	3.27	3.31	3.22
CG125	53251011	89.68	92	22	149	1.83	1.86	1.81
CG126	53251003	89.01	90	20	153	1.83	1.71	1.95
CG127	53250990	90.29	94	15	152	2.59	2.66	2.52
CG128	53250985	92.03	96	15	157	1.94	1.92	1.96
CG129	53250981	93.49	97	17	157	3.52	3.57	3.46
CG130	53250957	104.05	106	19	163	3.25	2.94	3.54
CG131	53250945	109.21	113	17	176	2.59	2.76	2.41
CG132	53250936	114.64	120	17	190	2.66	2.77	2.54

CG133	53250930	119.81	125	19	202	3.91	3.74	4.07
CG134	53250921	126.39	131	22	210	3.84	3.94	3.74
CG135	53250915	129.01	134	25	221	3.68	3.84	3.52
CG136	53250912	130.81	135	25	220	3.87	3.73	4.01
CG137	53250907	133.04	137	26	225	5.85	5.70	6.00
CG138	53250900	137.06	138	26	242	6.80	6.92	6.68
CG139	53250898	137.66	137	26	244	6.13	5.93	6.33
CG140	53250885	143.93	144	28	254	5.66	6.04	5.27
CG141	53250883	144.77	145	29	255	6.06	5.96	6.17
CG142	53250871	144.35	145	33	251	4.14	4.14	4.14
CG143	53250862	146.68	148	35	251	3.30	3.29	3.31
CG144	53250850	143.65	144	35	248	2.24	2.02	2.46
CG145	53250847	141.54	142	35	243	4.38	4.63	4.14
CG146	53250837	132.18	133	33	224	5.43	5.43	5.43
CG147	53250828	121.86	122	33	210	3.56	3.53	3.59
CG148	53250825	117.38	117	29	202	3.40	3.58	3.22
CG149	53250805	93.80	91	27	165	3.02	2.71	3.33
CG150	53250792	78.66	77	23	141	4.01	3.64	4.38
CG151	53250782	65.35	62	18	125	16.02	15.04	16.98
CG152	53250775	54.78	53	16	105	6.09	6.11	6.06
CG153	53250773	51.02	49	16	100	5.46	5.87	5.05
CG154	53250764	41.28	40	14	84	6.29	6.25	6.33
CG155	53250759	34.22	33	11	67	5.68	4.87	6.47
CG156	53250745	21.62	22	3	42	13.14	12.39	13.88
CG157	53250719	50.07	49	11	87	25.97	25.22	26.69
CG158	53250667	103.19	103	20	158	9.03	8.70	9.35
CG159	53250572	156.61	156	24	254	53.93	54.1	53.75
CG160	53250566	157.31	157	25	248	43.12	42.68	43.56
CG161	53250562	157.38	158	24	243	52.53	52.25	52.8
CG162	53250552	156.47	158	25	251	48.43	48.3	48.56
CG163	53250525	150.95	153	26	238	34.85	33.36	36.32
CG164	53250482	133.69	137	25	216	68.76	67.62	69.87
CG165	53250410	120.27	123	18	188	79.13	79.42	78.84
CG166	53250325	149.83	152	26	246	81.56	81.81	81.32
CG167	53250259	166.16	165	36	277	52.95	52.29	53.59
CG168	53250139	113.09	111	28	186	87.37	87.14	87.59
CG169	53249824	217.05	220	51	376	87.74	87.13	88.34
CG170	53249789	200.45	197	50	350	78.60	78.46	78.74
CG171	53249568	148.41	153	27	242	81.66	81.10	82.21
CG172	53249480	182.98	182	38	281	84.28	83.99	84.57
CG173	53248846	280.19	281	66	444	88.18	87.93	88.42
CG174	53248825	280.05	280	66	450	89.62	89.65	89.6
CG175	53248529	280.01	279	67	500	81.35	81.71	81.00
CG176	53248211	210.26	211	40	369	78.76	78.63	78.89
CG177	53247951	8.87	9	2	17	95.38	95.54	95.21
CG178	53247768	58.42	58	15	109	93.12	93.23	93.01

CG179	53247745	1	1	1	1	14.29	25.00	0
CG180	53247658	151.47	153	28	232	41.61	40.70	42.50
CG181	53247612	203.77	204	48	312	83.78	83.98	83.59
CG182	53247533	205.31	209	51	334	65.42	64.31	66.51
CG183	53247531	202.28	206	50	333	89.31	88.99	89.63
CG184	53247527	198.18	200	51	330	88.51	87.76	89.24
CG185	53247373	190.91	196	56	291	91.89	91.82	91.97
CG186	53247330	187.89	194	61	297	81.89	81.62	82.16
CG187	53246357	131.33	134	26	209	83.75	84.63	82.89
CG188	53246281	145.15	145	30	233	52.21	52.72	51.71
CG189	53246208	132.97	131	32	212	49.81	50.20	49.43
